# A Novel Nitrogen Ion Implantation Technique for Turning Thin Film “Normally On” AlGaN/GaN Transistor into “Normally Off” Using TCAD Simulation

**DOI:** 10.3390/membranes11110899

**Published:** 2021-11-20

**Authors:** Gene Sheu, Yu-Lin Song, Dupati Susmitha, Kutagulla Issac, Ramyasri Mogarala

**Affiliations:** 1 Department of Computer Science and Information Engineering, Asia University, Taichung 41354, Taiwan; g_sheu@asia.edu.tw (G.S.); smithadupati@gmail.com (D.S.); kutagullaissac23@gmail.com (K.I.); ramya3620@gmail.com (R.M.); 2 Department of Bioinformatics and Medical Engineering, Asia University, Taichung 41354, Taiwan

**Keywords:** TCAD, normally off, Nitrogen ion implantation, positive temperature coefficient

## Abstract

This study presents an innovative, low-cost, mass-manufacturable ion implantation technique for converting thin film normally on AlGaN/GaN devices into normally off ones. Through TCAD (Technology Computer-Aided Design) simulations, we converted a calibrated normally on transistor into a normally off AlGaN/GaN transistor grown on a silicon <111> substrate using a nitrogen ion implantation energy of 300 keV, which shifted the bandgap from below to above the Fermi level. In addition, the threshold voltage (V_th_) was adjusted by altering the nitrogen ion implantation dose. The normally off AlGaN/GaN device exhibited a breakdown voltage of 127.4 V at room temperature because of impact ionization, which showed a positive temperature coefficient of 3 × 10^−3^ K^−1^. In this study, the normally off AlGaN/GaN device exhibited an average drain current gain of 45.3%, which was confirmed through an analysis of transfer characteristics by changing the gate-to-source ramping. Accordingly, the proposed technique enabled the successful simulation of a 100-µm-wide device that can generate a saturation drain current of 1.4 A/mm at a gate-to-source voltage of 4 V, with a mobility of 1487 cm^2^V^−1^s^−1^. The advantages of the proposed technique are summarized herein in terms of processing and performance.

## 1. Introduction

Silicon power transistors have been developed for decades but have recently started facing physical limits in terms of mobility, high-voltage, and high frequency, preventing further major advancements [1]. The increase in input and output capacitance levels with decreasing *R_DS_* (on) hampers high-switching-frequency operation due to increased switching loss. By contrast, gallium nitride (GaN) transistors, which are characterized by considerably lower capacitance levels, are only at the infancy stage of development and their lower capacitance levels are due to their superior material and physical properties such as higher conductivity, higher energy gap, and higher electrical field as compared with silicon [2].

Growing GaN naturally on a silicon substrate results in a normally on or a D-mode device inherently characterized by stress-induced two-dimensional electron gas (2DEG) formation [3]. Because of their strong intrinsic or spontaneous charge polarization, conventional wide bandgap GaN-based heterojunction devices can deliver excellent performance in ultra-high frequency power amplifiers and high-voltage power devices [4,5,6,7,8,9,10,11,12]. Normally on devices can be designed and used in such applications, but these require a threshold voltage (V_th_) to turn off and are dangerous because once the power is switched off, the device turns on automatically. Thus, designers do not prefer normally on devices and are more inclined toward normally off devices. Normally off or E-mode devices are more commonly employed in power engineering owing to their safety and reliability. Nearly all normally off GaN transistors currently available on the market use gate doping to increase V_th_ and improve dynamic R_on_ [13]; determining an optimal trade-off between cost/performance and mass manufacturability remains a challenge [14]. 

Gate recess [15], fluorine plasma ion implantation [16], and p-type cap [17] techniques are the commonly used approaches for fabricating normally off High-Electron-Mobility Transistors (HEMTs). In this study, we developed an innovative technique that entails using nitrogen ion implantation along with an annealing process to turn a normally on transistor into a normally off one. We provide an overview of the underlying physical mechanism of the nitrogen ion implantation technique that affords a robust approach for fabricating normally off GaN HEMTs. This topic is important because GaN transistors are expected to be employed in an increasing number of power semiconductor devices in the next few years. The normally on attribute of HEMT devices can be attributed to the presence of 2DEG formation in GaN-based heterostructures [18]. Thus, developing reliable solutions for achieving normally off GaN HEMTs remains a challenge. Although normally on HEMTs can be used in power electronics applications by adopting the “cascade” configuration, the market demands “real” normally off transistors. Compared with other processes, nitrogen ion implantation is simpler, more cost-effective, and more suitable for mass manufacturing. N vacancies act as donors in GaN. Therefore, the nitrogen ion implantation technique can reduce the leakage current among neighboring devices by up to five orders of magnitude because it allows efficient fabrication. Nitrogen ion implantation can enable the fabrication of a normally off device with a quantum well that considerably exceeds the Fermi level; this can be attributed to two major properties of nitrogen implantation. First, in the nitrogen ion–implanted structure, the 2DEG channel conduction band (quantum well) is just over the Fermi level, indicating the formation of a depleted channel by the masking of all the electrons within the quantum well. Second, through nitrogen ion implantation, the stress-induced 2DEG charges in the GaN crystal can be blocked. Nitrogen ion implantation is a safe, environmentally friendly, and low-cost process that can create a P-type defect with a 0.59-V activation energy level below the conduction band.

Using the nitrogen ion implantation technique, we successfully converted a calibrated normally on transistor into a normally off transistor with a good IV family curve, adjustable threshold voltages, high gain, favorable mobility, and a breakdown voltage of 127.4 V at room temperature. The designed normally off transistor also showed a low leakage current.

## 2. Device Structure and Simulation Setup

The AlGaN/GaN heterostructure with nitrogen ion implantation starts with silicon <111> substrate of 2 µm thickness doped with P-type. Silicon substrate has better thermal conductivity when compared to the GaN substrate itself [19] and it is the best source for good conductivity and cost-effective process. This feature enables the integration of high-power electronic GaN on Silicon substrate that can have surface cracks and dislocations caused by in-plane lattice constant mismatch of 16.94% which can be lowered using AlN (Aluminum Nitride) layer [20]. The AlN layer has an in-plane lattice constant of a_0_ = 3.11 Å which is lower than that of GaN a_0_ = 3.18 Å. Therefore, an epitaxial layer grown on the AlN nucleation layer will reduce the tensile stress induced by a mismatch between GaN and silicon during the cooling process and avoid melt-back itching of GaN into Si.

**Figure 1:**
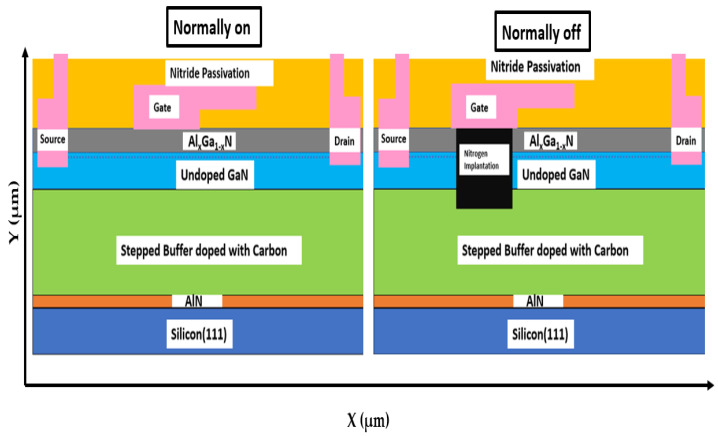
Schematic device structure of normally on and normally off AlGaN/GaN HEMT.

The AlN layer could also protect the epitaxial layer from wafer bowing and cracking. The epitaxial layer structure consisted of a 2-µm silicon substrate and a 3.9-µm-thick AlGaN/GaN/AlGaN/GaN buffer layer on which a 0.3-µm-thick undoped GaN layer was deposited. The stacked buffer layer was doped uniformly with a carbon concentration of 1 × 10^18^ cm^−3^. This concentration implies that the GaN buffer could be grown under low-pressure settings at a residual carbon-doping concentration. The selected carbon-doping concentration and stacked buffer layer thickness helped reduce the leakage current and improve the blocking voltage of the device. Furthermore, a 27-nm-thick Al_x_Ga_1−x_N barrier layer was deposited on top of the undoped GaN layer. A 2DEG channel region formed at the interface of the AlGaN barrier and GaN undoped layer. The calibrated normally on and the simulated normally off device structures are illustrated in Figure 1 [21,22,23].

The simulated GaN/AlGaN device was determined to have a thickness of <8 µm and a width of 100 µm, which was considered the area factor in the Sentaurus TCAD device simulator. The device dimensions are outlined as follows: gate length, 6 µm; source-to-gate distance, 4 µm; and gate-to-drain distance, 23 µm. The source and drain contacts were annealed at 900 °C for 25 s. To reduce the electric field through gate field plate optimization, a gate with a field plate length of 4.5 µm was used; this thus reduced the probability of current collapse. The entire GaN device structure was passivated with a nitride passivation layer. The device is in X-limits from 0 to 83 µm and Y-limits from o to 7.8 µm. Box method is obtained is used to obtain various mesh sizes for regions as 8 × 10^−1^ µm in substrate region, 1.6 × 10^−2^ µm in buffe region, 8 × 10^−3^ µm in channel region 1.6 × 10^−2^ µm in passivation region. The simulations were performed using the finite element software Synopsys Sentaurus simulator, device structure is obtained using Sentaurus process, which is a process simulator equipped with a set of advanced process models, which include default parameters calibrated with data from equipment vendors, Sentaurus Process provides a predictive framework for simulating a broad range of technologies from nanoscale CMOS to large-scale high-voltage power devices. The nitrogen ion implantation profile was obtained using the TRIM (Transport of Ions in Matter) simulator, which calculate the interaction of matter. The electrical and transfer characteristics are measured using Sentaurus Device which can simulate electrical, thermal, and optical characteristics of silicon-based and compound semiconductor devices and supports the modeling of high mobility channel materials and implements highly efficient methods for modeling. For simulation purpose the best models are selected from the TCAD simulation software. In Table 1, the models used for the simulation of current normally off AlGaN/GaN HEMT are listed. The key parameters for the precise tuning of threshold voltage (Vth) in GaN transistors are the control of the positive fixed charges −5 × 10^12^ cm^−2^, donor-like traps −3 × 10^13^ cm^−2^ at the nitride/GaN interfaces, the energy of the donor-like traps 1.42 eV below the conduction band and the acceptor traps activation energy in the AlGaN layer and buffer regions with 0.59 eV below the conduction band. 

## 3. Results and Discussion

In general, GaN transistors are normally on devices due to 2DEG formation. To convert them into normally off devices, the electrons in the 2DEG region must be partially blocked. If the implantation-induced vacancy concentration exceeds 1 × 10^18^ cm^−3^, the device would reach the isolation mode [24].

**Figure 2:**
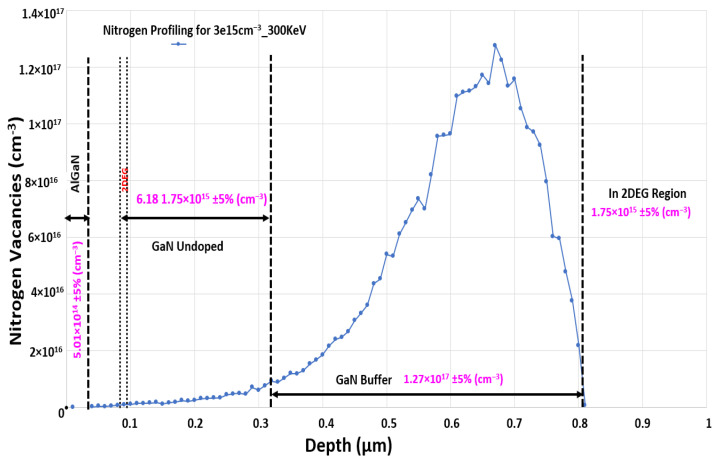
Nitrogen Implantation profile for AlGaN/GaN HEMT.

In this study, when the nitrogen ion implantation energy was 300 keV and dose was 3 × 10^15^ cm^−2^, the simulated device showed a vacancy concentration of 1.75 × 10^15^ cm^−3^ in the 2DEG region, which was sufficient to convert the device into a normally off one. Moreover, the device had a positive threshold voltage of approximately 0.5 V. The nitrogen vacancy concentration profile obtained using the TRIM simulator is displayed in Figure 2. 

**Figure 3:**
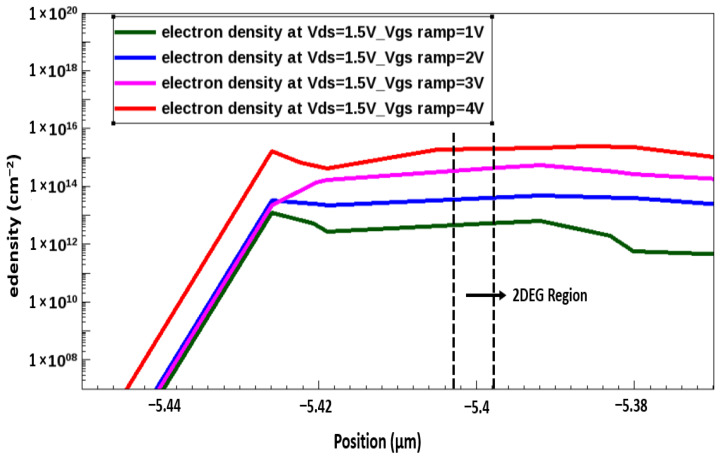
2 DEG density after nitrogen implantation for AlGaN/GaN HEMT.

In the GaN/AlGaN device, the quantum well formed at the interface of the AlGaN and undoped GaN layer. A quantum well confines electrons in normally on devices because it is typically below the Fermi level; however, in normally off devices, the quantum well is typically above the Fermi level [25]. Using the nitrogen ion implantation technique, we obtained a normally off device for which the quantum well was above the Fermi level by 0.8 eV. This can primarily be attributed to two major properties of the nitrogen ion implantation technique. First, in the implanted structure, the 2DEG channel conduction band (quantum well) is just above the Fermi level, indicating the formation of a depleted channel through the masking of all the electrons in the quantum well. Second, the negatively charged N ions cause an upward bending of the conduction band, especially in the AlGaN barrier, yielding an additional barrier height and resulting in a suppression of the gate leakage [26,27,28]. The 2DEG electron densities observed for the device before and after nitrogen implantation are presented in Figure 3. As indicated in this figure, the 2DEG density could be partially blocked at an implantation energy of 300 keV, and the gate voltage could finely adjust the 2DEG density.

**Figure 4:**
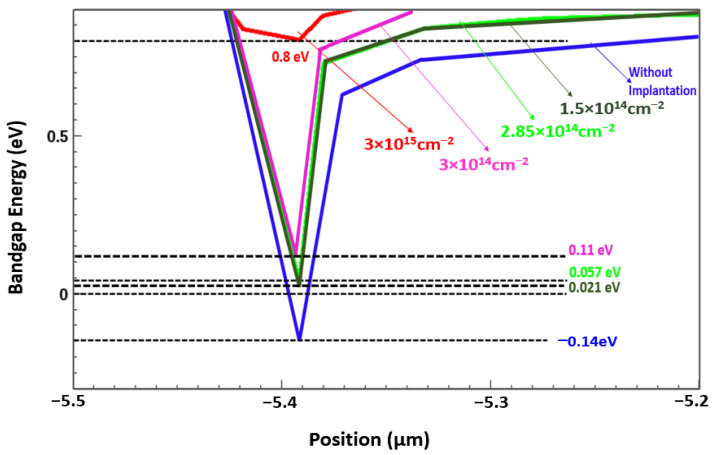
Bandgap analysis of AlGaN/GaN with nitrogen ion implanted gate HEMT.

**Figure 5:**
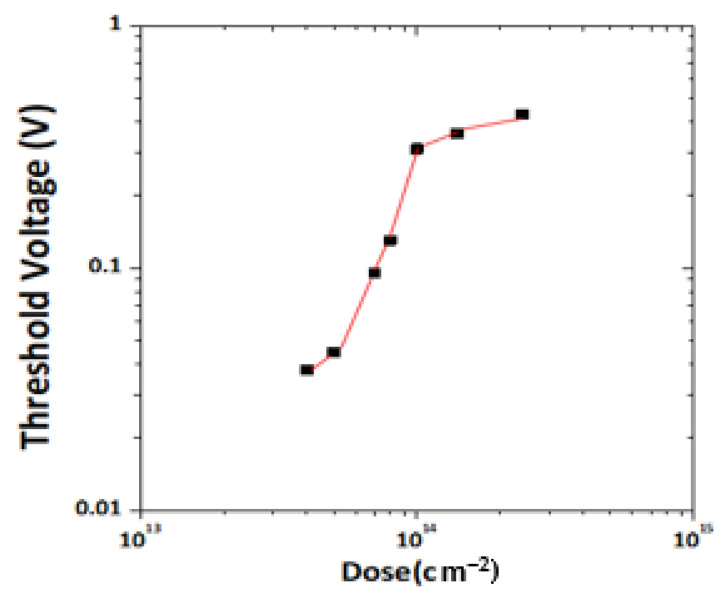
Trend Curve for Dose vs. Threshold Voltage.

The key to normally off HEMT technology is the localized conversion of device properties from normally on to normally off through threshold voltage control. In this study, the position of the quantum well shifted as the implantation dose changed (Figure 4); therefore, the threshold voltage (V_th_) shifted for which the trend curve of dose vs threshold voltage is presented in Figure 5. 

**Figure 6:**
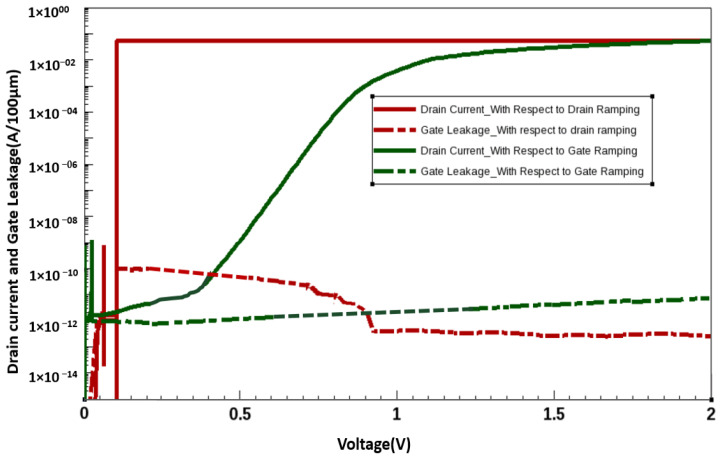
IV Curve with gate ramping = 2 V for AlGaN/GaN HEMT.

**Figure 7:**
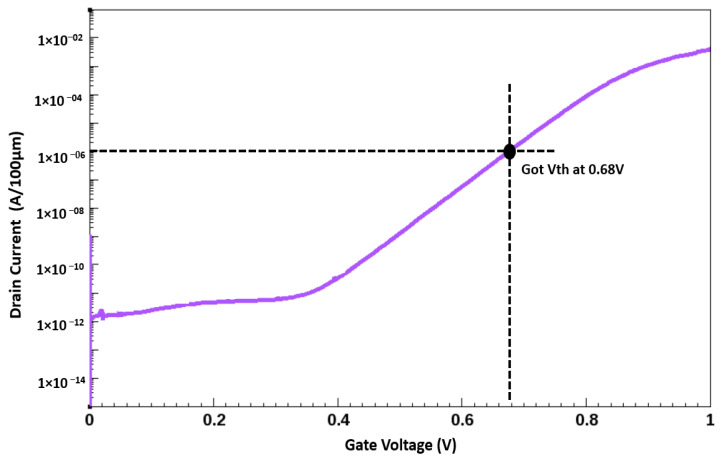
Vth Curve for AlGaN/GaN with nitrogen implanted gate HEMT.

**Figure 8:**
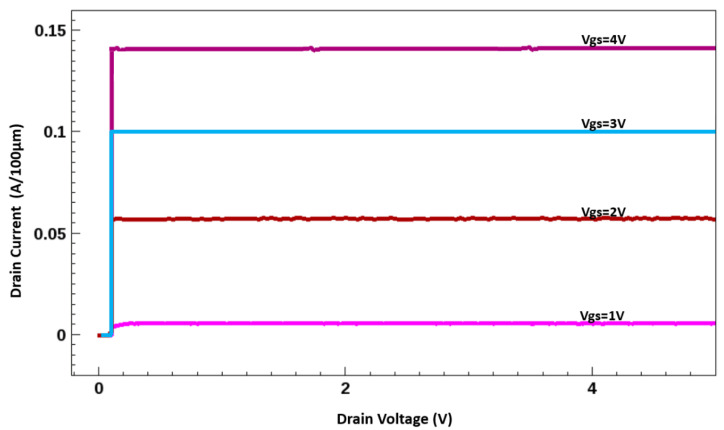
IV Curve with different Vgs ramping AlGaN/GaN HEMT.

The I–V characteristics of the simulated AlGaN/GaN HEMT with ramping of Drain Voltage (V_d_) = 0.1 V at Gate Voltage (V_g_) = 0 V then V_g_ = 2 V at V_d_ = 0.1 V and then V_d_ = 10 V at V_g_ = 2 V, with the implanted gate are illustrated in Figure 6, indicating a small leakage current. The simulated AlGaN/GaN HEMT with the implanted gate exhibited a threshold voltage (V_th_*)* of 0.68 V (Figure 7). The threshold voltage (V_th_*)* for the data are considered at the drain current of 1 mA/mm and the ramping conditions are V_d_ = V_g_. The device exhibited a saturated drain current of 1.43 A/mm at V_GS_ = 4 V and V_DS_ = 10 V. The saturation current characteristics observed at various gate voltages (1–4 V with a step of 1 V) are displayed in Figure 8. The I–V curves show an average gain of 45% per gate voltage.

**Figure 9:**
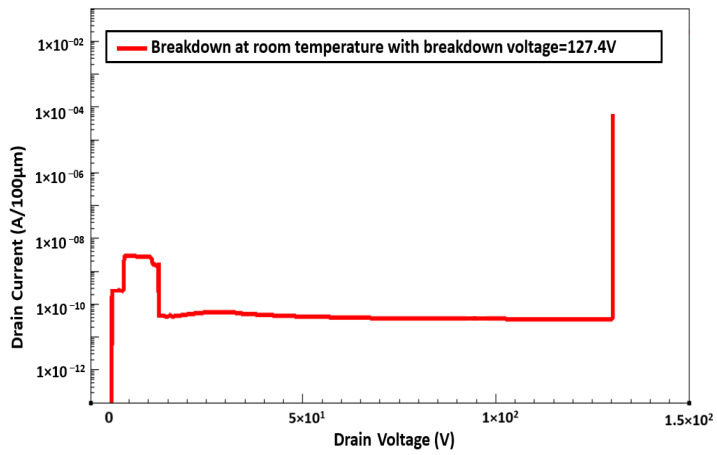
Breakdown Curve at room temperature.

**Figure 10:**
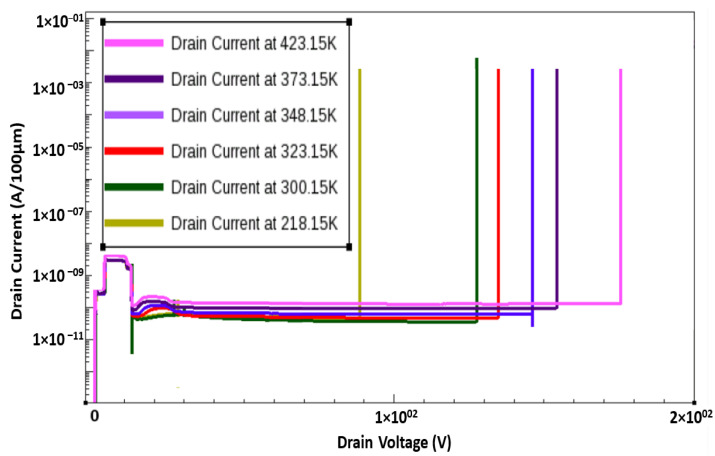
Temperature-dependent breakdown curve for a normally off AlGaN/GaN HEMT.

Impact ionization in the channel was triggered by the gate electrons, which resulted in the breakdown of the device. During the breakdown, a positive temperature coefficient was observed after the calculation of the temperature dependence of breakdown voltage. This is because the mean free path of electrons, which is limited by phonon scattering, is shorter at high temperatures [29]; thus, a higher electric field is required to gain the energy required for impact ionization. By contrast, during the surface breakdown of the device, a calculation of the temperature dependence of breakdown voltage revealed a negative coefficient. This is because the main mechanism of electron transport through surface states is hopping conduction, which is significant at high temperatures. We successfully simulated a normally off device with a breakdown voltage of 127 V at room temperature (Figure 9). The temperature dependence of the breakdown voltage is shown in Figure 10.

**Figure 11:**
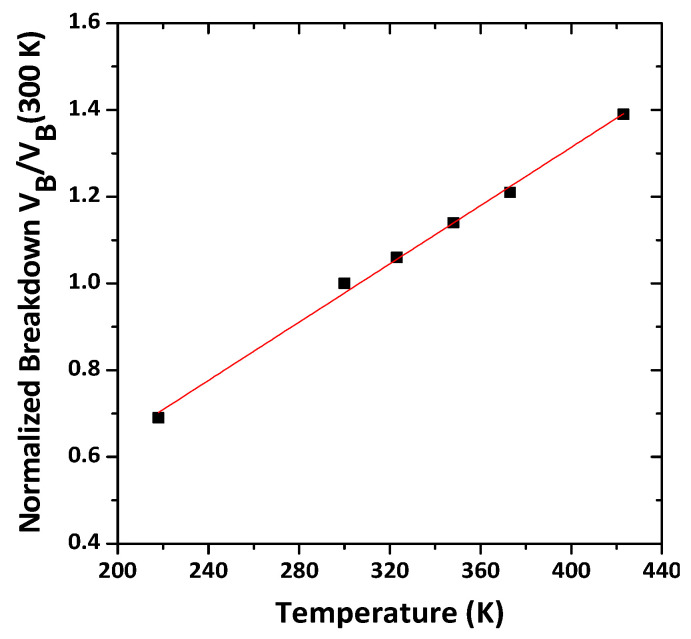
Temperature vs. Normalized Breakdown.

The temperature dependence of breakdown voltage can be expressed as follows [30]:

BV(T) = BV_300K_ (1 + *k*ΔT) (1)

where *k* is the temperature coefficient. The measured coefficient for breakdown voltage was 3 × 10^−3^ K^−^^1^. The measured temperature-dependent breakdown voltage of the GaN HEMT and the corresponding temperature coefficient were in good agreement with the results reported in [31]. The plot of temperature versus normalized breakdown is illustrated in Figure 11.

## 4. Conclusions

In this study, we developed a low-cost mass-manufacturable ion implantation technique for converting thin film normally on AlGaN/GaN transistors into normally off ones using TCAD simulation. The normally off AlGaN/GaN HEMT is achieved by partially masking the 2DEG using the nitrogen ion implantation technique. We observed that the threshold voltage can be tuned by varying the nitrogen implantation dose. The achieved normally off transistor exhibited good I–V characteristics, with a measured drain current gain of 45.3% and a low leakage current. Moreover, we found that the breakdown was caused by impact ionization. The temperature-dependent curve showed a positive temperature coefficient of 3 × 10^−3^ K^−1^. Furthermore, we demonstrate the processing and performance of the nitrogen ion–implanted thin film normally off transistor.

## Figures and Tables

**Table 1:** Models used in GaN device simulation.

Physical Phenomenon	Model
Mobility	1a. Doping dependence
	1b. High field saturation
	1c. Poole Frankel
Avalanche	2a. Van overstraeten
Recombination	3a. Shockley-Red-Hall
Polarization	4a. Piezo-Electric Stress
	4b. Piezo-Electric Strain
Tunneling	5a. Electron Barrier Tunneling
Self-heating effect	6a. Thermodynamic

## Data Availability

Not applicable.
